# Personals

**Published:** 1918-02

**Authors:** 


### PERSONAL

Dr. A. F. Soch of Fredonia narrowly escaped serious injury when his auto skidded into a Valley Branch train which was crossing the main street of the town.

The "Nurse" has been taken over by the "Trained Nurse and Hospital Review" beginning with the December issue.

Dr. Jacob E. Helwig of N. Tonawanda has succeeded Dr. Henry Smoyer as Health Officer of the town of Wheatfield, Dr. Smoyer having recently left for Porto Rico.

Dr. M. E. Babcock of Bath, (Univ. of Buffalo 1884), has had a series of misfortunes. On Oct. 26, late at night, he was knocked down by an auto and rendered unconscious, requiring treatment at the Bath Hospital before being removed to his home. Just as he was able to begin work again, he contracted diphtheria from a patient on Nov. 8, and was not released from quarantine till Dec. 28. He is still lame from his first accident, though no bones were broken and has not fully recovered from the combined shock of the accident and the infection. Dr. Babcock has our sincere sympathy and best wishes for a rapid recovery.

Dr. Hideo Noguchi of N. Y. was operated on for appendi citis, Dec. 19.

Dr. B. D. Dhedd of Arcade has been elected junior warden of the local Masonic chapter.

